# Experimental tissue mimicking human head phantom for estimation of stroke using IC-CF-DMAS algorithm in microwave based imaging system

**DOI:** 10.1038/s41598-021-01486-x

**Published:** 2021-11-10

**Authors:** Mohammad Shahidul Islam, Mohammad Tariqul Islam, Ali F. Almutairi

**Affiliations:** 1 Department of Electrical, Electronic and Systems Engineering, Faculty of Engineering and Built Environment, Universiti Kebangsaan Malaysia, 43600 Bangi, Selangor Malaysia; 2 Electrical Engineering Department, College of Engineering and Petroleum, Kuwait University, 13060 Safat, Kuwait

**Keywords:** Biomedical engineering, Electrical and electronic engineering

## Abstract

This paper presents the preparation and measurement of tissue-mimicking head phantom and its validation with the iteratively corrected coherence factor delay-multiply-and-sum (IC-CF-DMAS) algorithm for brain stroke detection. The phantom elements are fabricated by using different chemical mixtures that imitate the electrical properties of real head tissues (CSF, dura, gray matter, white matter, and blood/stroke) over the frequency band of 1–4 GHz. The electrical properties are measured using the open-ended dielectric coaxial probe connected to a vector network analyzer. Individual phantom elements are placed step by step in a three-dimensional skull. The IC-CF-DMAS image reconstruction algorithm is later applied to the phantom to evaluate the effectiveness of detecting stroke. The phantom elements are preserved and measured multiple times in a week to validate the overall performance over time. The electrical properties of the developed phantom emulate the similar properties of real head tissue. Moreover, the system can also effectively detect the stroke from the developed phantom. The experimental results demonstrate that the developed tissue-mimicking head phantom is time-stable, and it shows a good agreement with the theoretical results in detecting and reconstructing the stroke images that could be used in investigating as a supplement to the real head tissue.

## Introduction

Brain stroke is one of the most frequent causes of death and disability in developed countries as well as developing countries. It is noteworthy that worldwide each year, ap-proximately 16 million people are affected by stroke, of which 6 million die and another 6 million eternally become disabled. According to the united states (US) stroke statis-tics, more than 142,000 people pass away from stroke, about 796,000 people suffer a stroke, as well as approximately 650,000 of these are initial attacks and 187,000 are recur-rent attacks each year^1^. However, a stroke is characteristically categorized as a neuro-logical discrepancy accredited to a severe crucial injury of the vital nervous system by a vascular cause. Most strokes are caused by some specific issues, such as (i) sudden interruption in the blood supply of the brain, (ii) an abrupt blockage of arteries leading to the brain, and (iii) bleeding into brain tissues when a blood vessel bursts. Due to these incidences, it not only hampers the important functionality of the brain but also causes a fatality. Nowadays, Magnetic resonance imaging (MRI), computed tomography (CT), and positron emission tomography (PET) imaging are the existing popular technologies in the medical diagnostic imaging system to detect brain stroke in the human head. Although these existing technologies are highly sensitive in terms of brain stroke diagnosis, they are very much expensive, and not easily accessible and affordable for the patients of rural hospitals^2^. According to the World Health Organization (WHO), reliable and affordable medical imaging systems are not accessible to about three-quarters of the world population^3,4^. Furthermore, these existing imaging systems are very bulky which is not easily carried out by the clinical staff for the diagnosis purpose^5,6^. Hence, a portable, non-ionizing, low-cost imaging system is owing popularity for the detection of brain stroke. Microwave imaging has been introduced as a complementary rapid, non-ionizing, lightweight, low-cost, and low-profile technique to that stated typical existing medical diagnostic methods^7–9^.

The human head phantom is the artificial replica that emulates the electrical properties of head tissue where the anatomical geometry is highly required for the verification of electromagnetic (EM) imaging system. These tissue-mimicking properties of the head phantom help to verify the effectiveness of the EM imaging system before starting the human trials. An anatomically reconfigurable head phantom is presented at^10^ for stroke detection. Mixtures of polyurethane rubber, graphite powder, and carbon black powder have been used to mimic the scalp, skull, and cerebrospinal fluid (CSF). The brain phantom is in liquid form to facilitate the insertion of stroke. However, a homogeneous medium is used to fabricate the head phantom, and due to the emulating liquid medium, the organs are randomly simplified for fabrication. A head mimicking phantom is presented at^11^, where the brain and blood have been fabricated for the imaging system. However, the phantom indicates the homogeneous characteristics, which does not properly reflect the tissue emulation. Besides, there are no experimental measurements on the electrical properties of fabricated materials. A 3D printed head model is presented at^12^, where the ABS materials have been used for fabrication. Nevertheless, the phantom properties do not show the heterogeneous characteristics that emulate the tis-sue-mimicking phantom. An experimental head phantom is proposed at^13^, where the phantom consists of CSF, brain, and blood components. However, this fabricated phantom does not properly reflect the tissue-mimicking characteristics due to the only consideration of main tissues in a layered structure. A head phantom containing polymer com-position materials is presented at^14^. Gray matter, white matter, and blood mimicking materials have been fabricated for EM head imaging. However, there is no justification of the fabricated phantom that can detect the stroke. Moreover, the casting steps of the fabricated materials are very much complex in terms of operational simplicity. Therefore, there is a need for fabricating a tissue mimicking head phantom that extensively emulates all the electrical properties of real head tissue.

**Figure 1:**
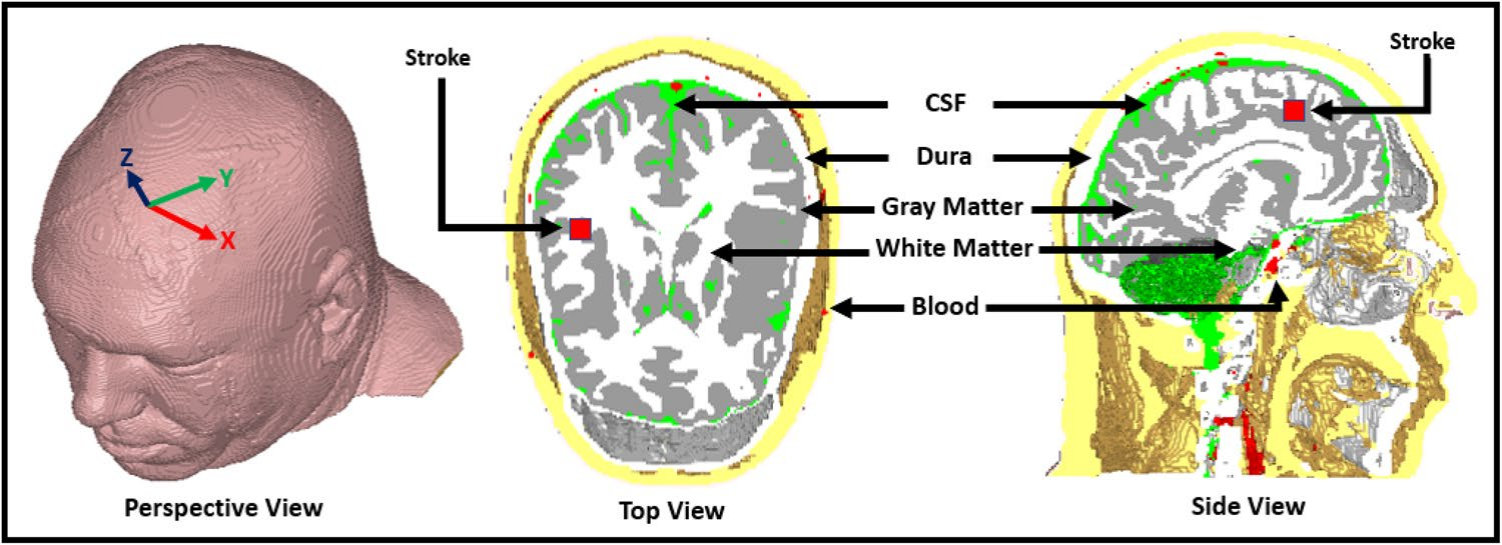
Layout of human head (Hugo model).

In EM head imaging system, antenna plays a key role for efficient and significant da-ta acquisition from the head phantom. In this technique, an array of antenna elements covers the surrounding area of the head and operates at single or multiple microwave frequencies. By analyzing and comparing the measured scattered data from these antenna array elements, the EM imaging solves the nonlinear inverse scattering problems by estimating the complex dielectric profile of the head domain. Tissue sensing adaptive radar and confocal imaging are the data independent algorithm that are used in imaging ap-plication; however, inverse scattering technique like delay and sum (DAS) is popular due to its operational simplicity and implementation^15–17^. DMAS, CF-DAS, and several other algorithms have been proposed recent years to improve the performance of DAS^18–22^. Other techniques like Born approximation is used to detect the scattering objects that do not alter the incident field significantly. Nevertheless, the multiple reflections are not analyzed in this method that makes the structure complex with many scattering interfaces. A Method like Distorted born iterative method (DBIM) has also been ap-plied to identify the scattering objects^13,23–25^. Yet, this technique often becomes unstable and diverge, especially in high noise environments. In this paper, a tissue-mimicking head phantom is prepared and fabricated, which emulates the real human head tissue. The electrical properties of the phantom elements are measured over time to evaluate the effectiveness of the tissue-mimicking materials. The modified Iteratively corrected coherence factor delay-multiply-and-sum (IC-CF-DMAS) algorithm is applied to reconstruct the stroke images. The IC-CF-DMAS algorithm enhances the computation for more accurate and precise stroke images. It is also data independent that makes it stable in a noisy environment. The algorithm is also applied to the phantom materials over the time to assess the stroke identification in EM head imaging system. Figure 1 represents the layout of the human head model. The model consists of CSF, dura, gray matter, white matter, and blood (represented as stroke). The next sections of this paper are organized as follows: “Phantom preparation and fabrication” section presents the phantom preparation and fabrication process, “Measurement and calibration techniques” section presents the measurement and calibration technique, “Dielectric properties of the phantom elements” section represents the dielectric properties of the measured phantom elements, “Data acquisition and image reconstruction” section reconstructs the image and validate with the fabricated phantom, and finally, “Stability in time” section presents the sensitivity analysis with the dielectric and reconstructed image changes over time.

## Phantom preparation and fabrication

Different compositions of sterile water, corn flour, gelatin, agar, Sodium Azide (NaN3), Propylene Glycol, and Sodium Chloride (NaCl) are used to fabricate the head phantom com-ponents of dura, CSF, gray matter, white matter, and blood or stroke. The composition is summarized in Table 1. The fabrication process starts with adding propylene glycol into sterile water in a small beaker for all the phantom components. The quantity is used as per the stated Table 1, and all the processes are performed in room temperature. Then the corn flour is added with the mixture by stirring gradually in small portions to make it a thick gelatinous syrup (component 1). Water is used as a main source of permittivity due to its high dielectric properties over the wideband frequency range. Propylene glycol is add-ed as a humectant and stabilizing agent, where it also helps to preserve the tissue by lowering the freezing temperature. Besides, the corn flour increases the thickness of the mixtures so that there is a strong bond created among the different layers of the tissue. In an-other beaker, agar is added to the sterile water and heated gradually up to 90–95℃. NaCl is then added to the mixtures when the temperature rises to that point, and the heating process is continued for another 3–4 min approximately until the agar is melted (component 2). This procedure with agar is applicable for fabricating Dura, CSF, gray matter and blood.

**Table 1:** Composition of the head phantom components.

Components	Dura (500gm)	CSF (500gm)	Gray matter (500gm)	White matter (500gm)	Blood/stroke (100gm)
Sterile water (gm/ml)	361.90	418.75	403.25	353.35	81.97
Corn flour (gm)	120.65	10.15	82.95	134.30	2.73
Gelatin (gm)	0.00	0.00	0.00	7.05	0.00
Agar (gm)	4.58	56.20	5.2	0.00	12.75
Sodium azide (NaN3) (gm)	1.8	1.85	1.75	1.75	0.36
Propylene glycol (gm)	9.65	7.45	4.6	3.55	0.91
Sodium chloride (NaCl) (gm)	1.20	5.60	2.30	0.00	1.28

**Figure 2:**
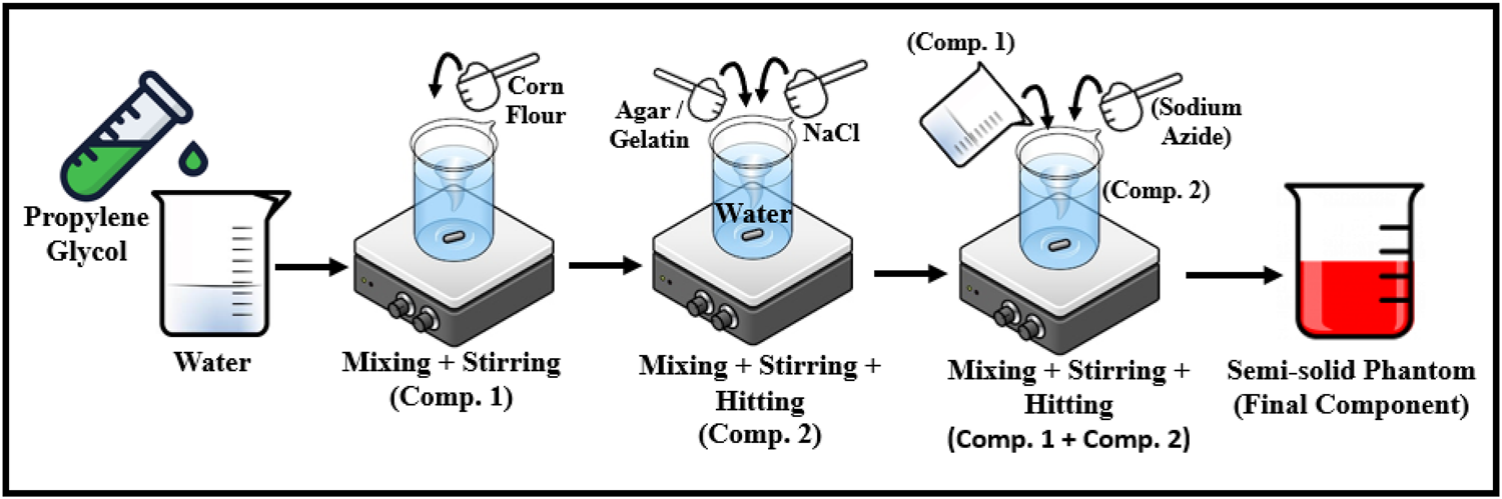
Schematic representation of the semi-solid phantom preparation process.

**Figure 3:**
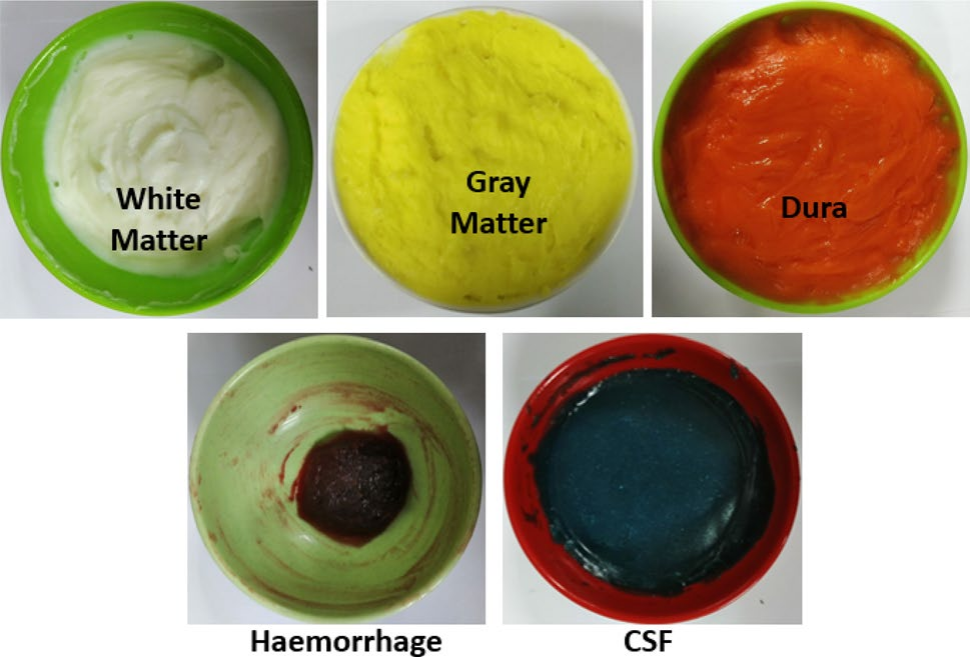
Fabricated components of the head phantom.

The gelatin is used alternatively for fabricating the white matter in a same procedure except using NaCl. NaCl controls the conductivity of the mixtures. The agar and gelatin control the relative permittivity and help to form semi-solid structure of the final tissue. The viscous syrup from the component 1 is added to the mixtures of component 2 where the burner and stirring process is kept running until the whole mixture becomes nearly semi-solid. Finally, the heating is stopped, and the mixture is allowed to cool down to 40–50℃. At this point, NaN3 is added as a preservative to the mixture, and the mixture forms into a semi-solid structure. The overall schematics of fabricating the phantom materials are depicted in Fig. 2. The materials used in fabricating the phantom components possess high mechanical properties and make it easy to produce the head phantom by considering different components in different layers. The fabricated components are presented in Fig. 3, where they are measured in the next step to validate the electrical properties.

## Measurement and calibration techniques

**Figure 4:**
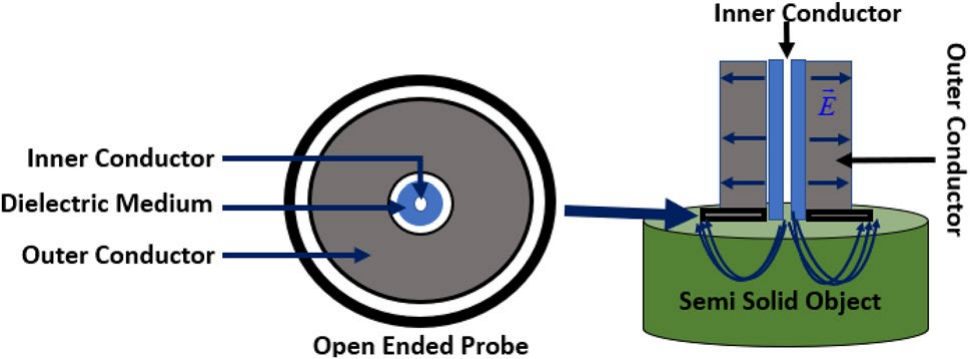
Cross-section schematics of the probe with electric field orientation.

The open-ended coaxial probe technique is used to measure the dielectric properties of the fabricated tissue-mimicking head phantom. This technique is simple, non-destructive, and applicable for both in-vivo and ex-vivo measurements over a broad frequency range. However, the limitations towards accurate measurements are observed because of the complex heterogeneous structures and uneven surface in the homogeneous structures. Calibration procedures and measurement devices like vector network analyzer (VNA) are the key factors that limit the measuring contents. The standard calibration procedure is applied with the three most common standards consisting of the open circuit, short circuit, and a broadband load while the probe (KEYSIGHT 85070E) is connected directly with the VNA (PNA-L N5232A; 300 kHz to 20 GHz). The aim is to correct the post-calibration measurement and make it reliable by analyzing the relation between the measured complex reflection coefficient and the expected one. Besides, environmental factors like temperature, pressure, and humidity, and system components like the cleanliness of the probe tip should be considered for a reliable measurement. Figure 4 represents the cross-section schematics of the used coaxial probe with its electric field orientation. The probe consists of a truncated section of transmission line where the EM waves propagate through the coaxial line. The impedance mismatch be-tween the probe and targeted tissue sample generates reflected signals, which are later converted into complex permittivity values.

**Figure 5:**
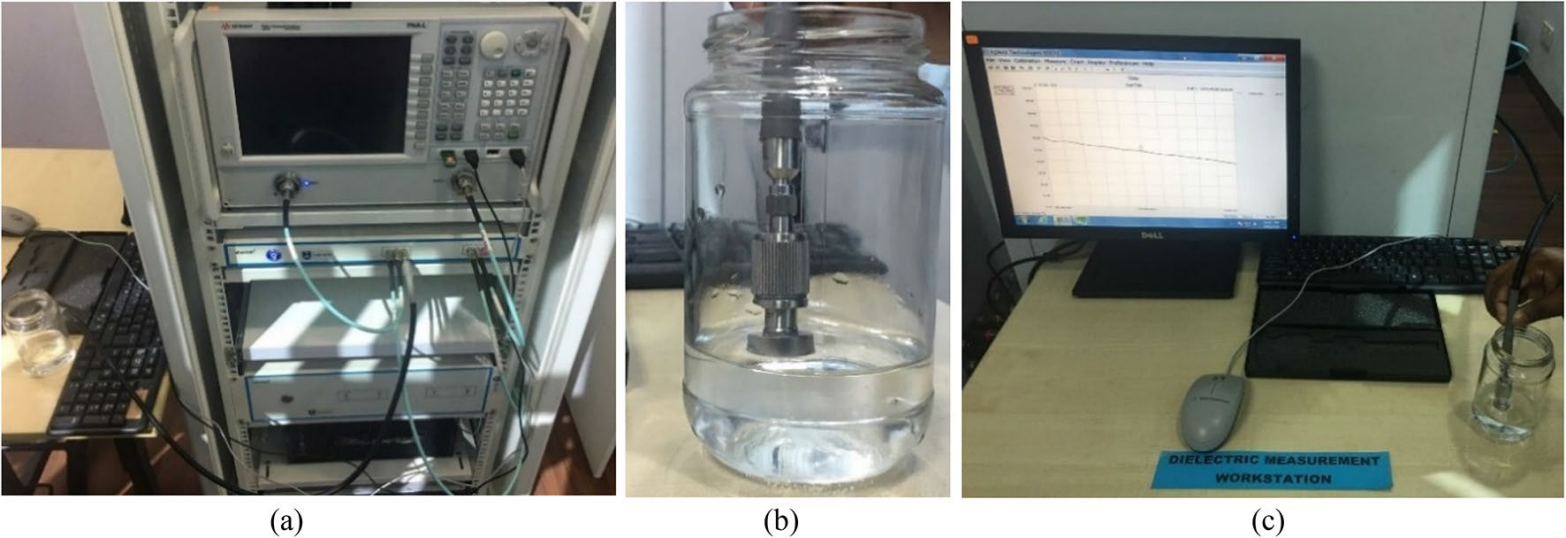
(**a**) Measurement setup with VNA (**b**) open-ended coaxial probe (**c**) calibration with sterile water.

**Figure 6:**
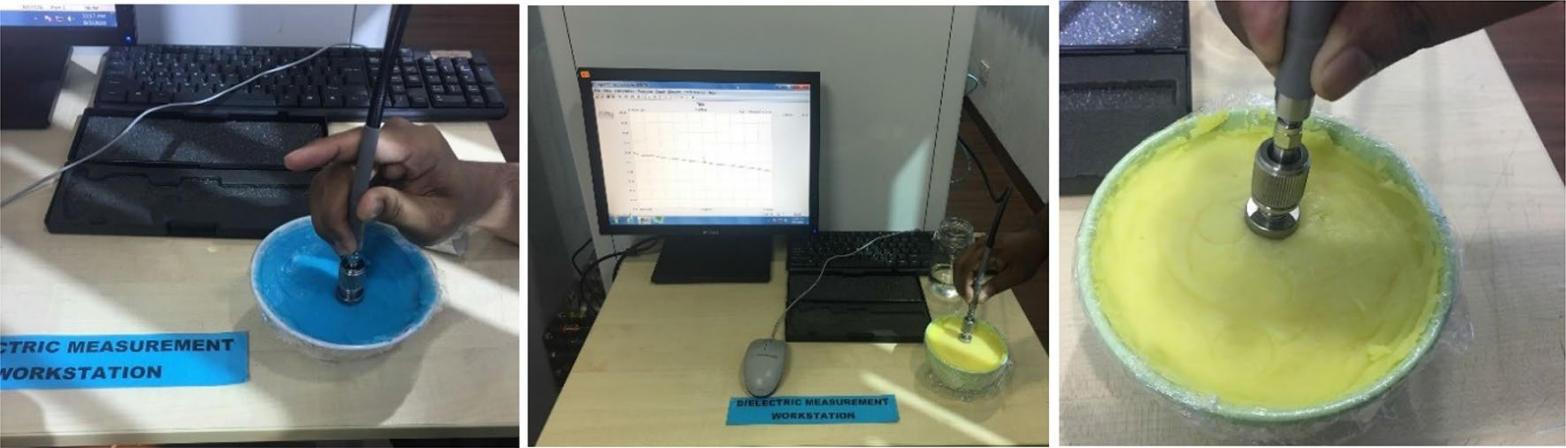
Measurement setup of the phantom components.

The vector network analyzer converts the reflected signal into complex permittivity. Figure 5 represents the starting phase of calibration with the 25 cm3 sterile water using the VNA and coaxial probe. Next, all the samples of the head phantom are sliced separately to ensure enough contact between the sample and the coaxial probe while performing the measurements. The visual inspection of the inner and outer part of the sample is analyzed to assess the consistency as the outer part is polished flat for ensuring no gap between the coaxial probe and sample component. The coaxial probe is placed randomly multiple times on the sample surface to collect the data for better accuracy. The final decision is then made by calculating the mean value from the taken multiple data. The measurement setup of the phantom components is depicted in Fig. 6. It is observed that the measurement is done as accurately as the average per-centage error is less than 2%.

## Dielectric properties of the phantom elements

**Figure 7:**
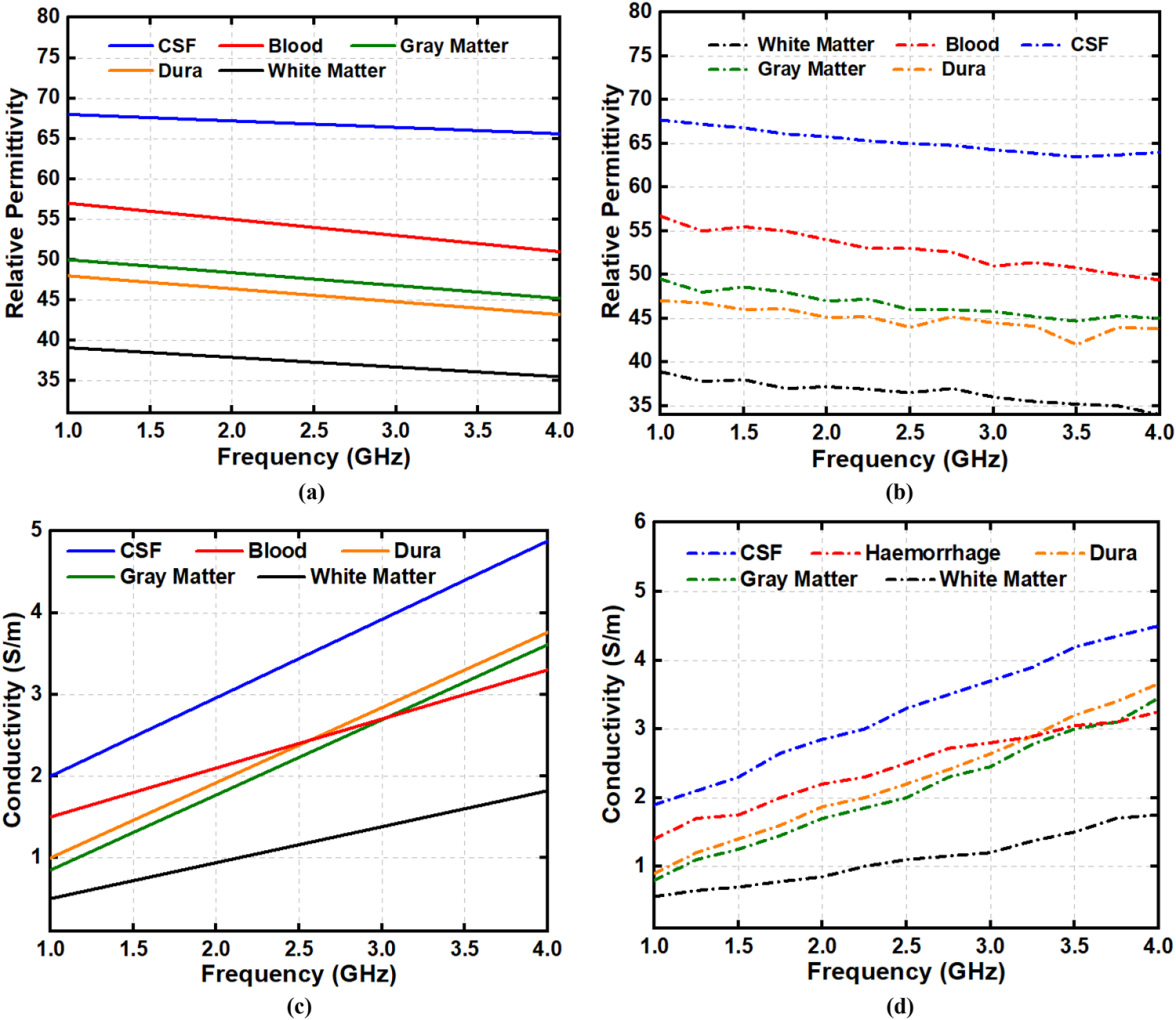
(**a**) Reference and (**b**) measured permittivity; (**c**) reference and (**d**) measured conductivity.

**Figure 8:**
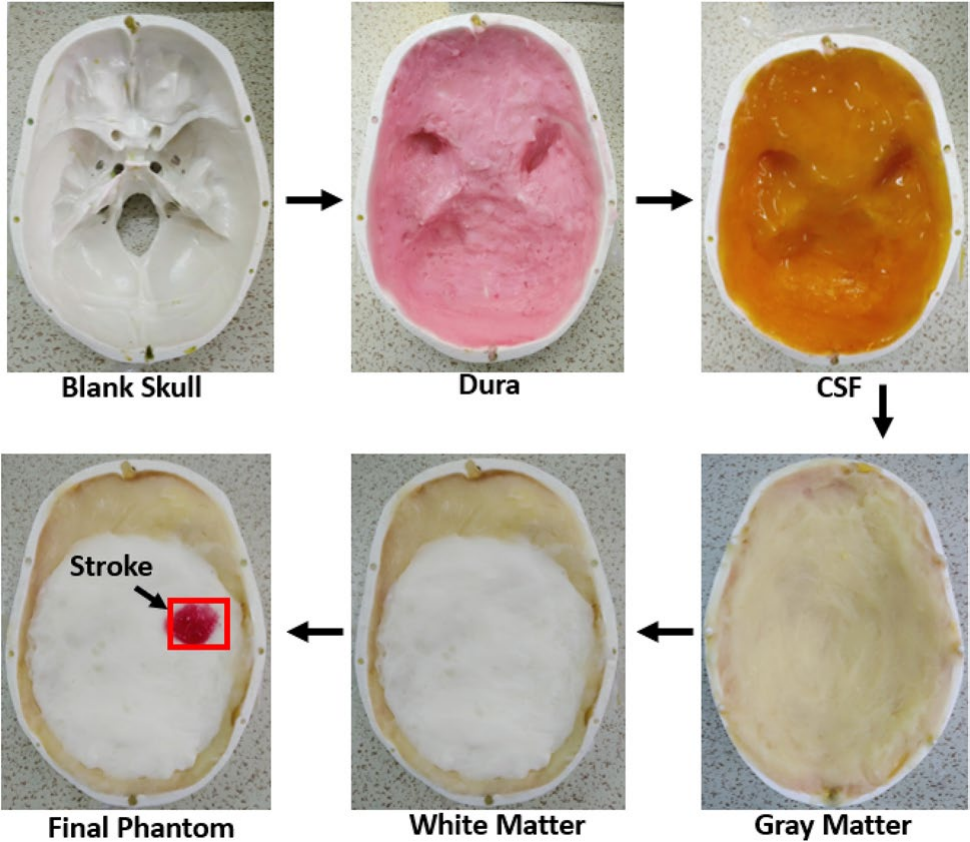
Overall placement process of the phantom layers.

Dielectric properties of the fabricated head phantom components are measured and compared with the reference relative permittivity and conductivity, depicted in Fig. 7. It is noticeable from the first measurement that the relative dielectric permittivity of the CSF, dura, gray matter, white matter, and blood range from 68–63, 48–42, 50–45, 40–34, and 57–49, respectively. The conductivity of the mentioned consecutive phantom components ranges from (in S/m) 1.9–4.5, 0.9–3.6, 0.8–3.3, 0.5–1.8, and 1.5–3.2, respectively. The open-ended coaxial probe is placed at two more random positions to evaluate the effectiveness of the electrical properties with the components. Table 2 represents the lists of data and its mean value which are taken from multiple positions of the phantom components at the centre frequency of 1 to 4 GHz. The relative permittivity reference values range from 68–60, 47–43, 50–45, 40–35, and 58–50, for the CSF, dura, gray matter, white matter, and blood, respectively. The conductivity values (in S/m) are 2–4.8, 1–3.8, 0.9–3.5, 0.5–1.8, and 1.5–3.2, respectively, for the stated consecutive components. It is observable that the relative permittivity and conductivity values are almost identical for the measured and reference parameters. Besides, the calculated mean value of relative permittivity and conductivity also show identical results comparing to the reference values. Therefore, the measured phantom shows more realistic characteristics of real human head tissue that evaluates the effectiveness of the electromagnetic head imaging system. The step by step adding procedure of the phantom components into a 3D skull is depicted in Fig. 8. Dura is the first component to be filled into it, which later continues with CSF, gray matter, white matter and blood, respectively. The rectangular mark represents the area of the stroke inside the head phantom, which is later analyzed through the IC-CF-DMAS image reconstruction algorithm to validate the phantom performance.

**Table 2:** Electrical properties of the fabricated head phantom from 1 to 4 GHz.

	CSF				Dura			
Properties	Data 1	Data 2	Data 3	Mean	Data 1	Data 2	Data 3	Mean
Permittivity, εr	65.15	61.26	58.33	61.58	44.04	41.35	42.56	42.65
conductivity (S/m)	3.26	2.78	3.45	3.16	2.15	1.84	2.34	2.11

	Gray matter				White matter			
Properties	Data 1	Data 2	Data 3	Mean	Data 1	Data 2	Data 3	Mean
Permittivity, εr	46.32	41.62	39.85	42.59	36.73	31.54	33.84	34.04
conductivity (S/m)	2.06	1.88	2.24	2.06	1.15	0.95	1.23	1.11

	Blood/stroke							
Properties	Data 1	Data 2	Data 3	Mean				
Permittivity, εr	52.81	49.65	55.87	52.78				
Conductivity (S/m)	2.55	2.78	2.12	2.48

## Data acquisition and image reconstruction

**Figure 9:**
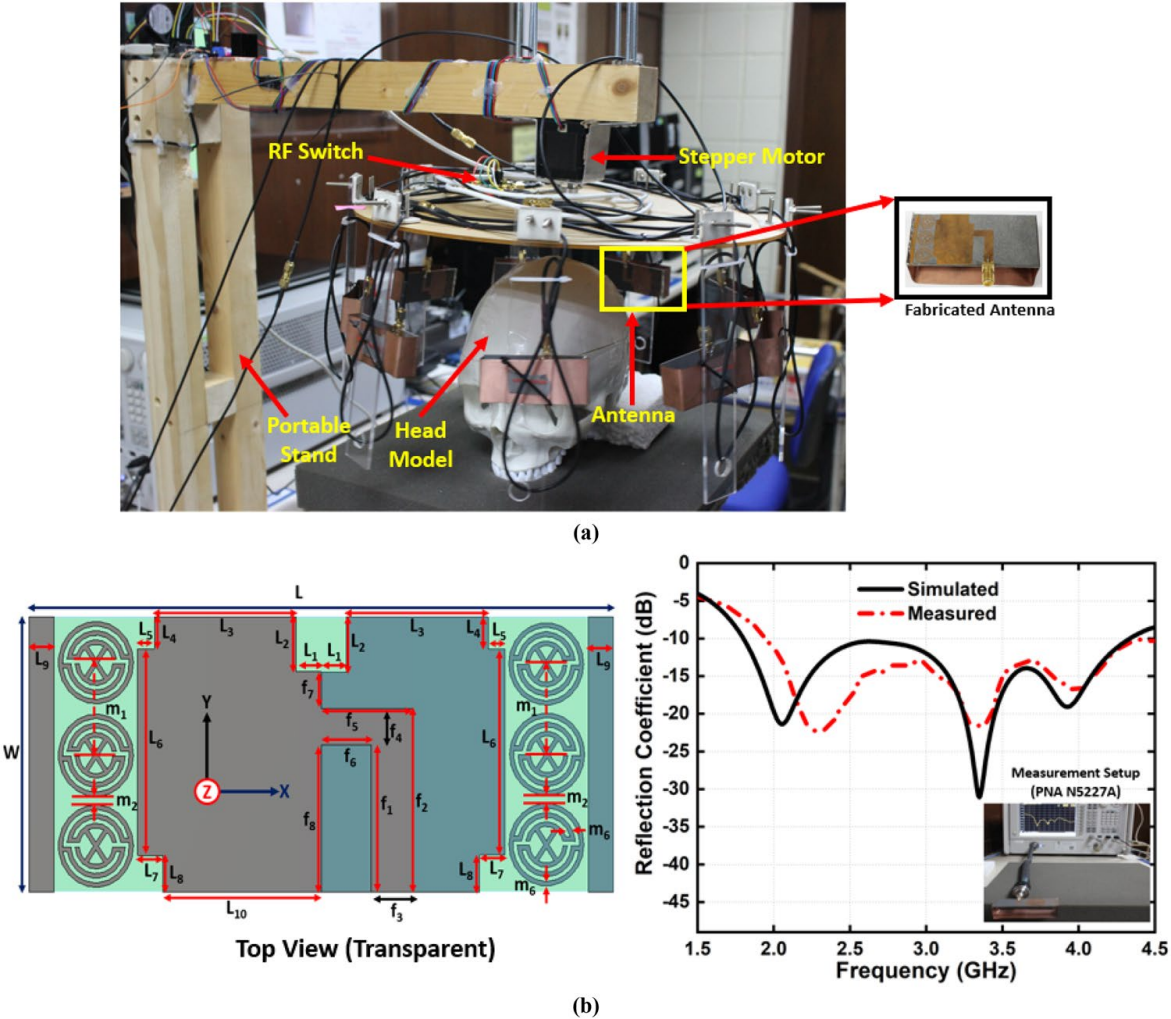
**(a)** Imaging system setup with fabricated antenna (**b**) Schematic profile of antenna and its reflection coefficient.

The head model with fabricated phantom will be placed in the centre of a nine-antenna array system where one antenna acts as a transmitter and others act as receivers. A stepper-motor based mounting stand, an SP8T RF switching system, and a personal computer-based image processing unit are also combined with the system. Nine transparent plastic sticks with antennas are installed on the rotating platform. The mechanical rotation platform rotates in polar coordinates from 0 to 2π around it. The data (S21, S31, S41, S51, S61, S71, and S81) are collected at each 7.2°, and 50 equal points, covering the total 360°. The Agilent E8358A Power network analyzer (PNA) works as a transceiver that generates EM signals through the transmitting antenna. This PNA is also connected to the personal computer via GPIB port that processes the received data for image reconstruction. The updated Iterative Correction of Coherence Factor Delay-Multiply-and-Sum (IC-CF-DMAS) algorithm is analyzed based on microwave signal contrast where the scattered EM signals from healthy and unhealthy head phantoms are compared. This comparison takes place between the reference microwave signal using numerical simulation of full-wave time-domain and the scattered signal from the computational phantom. A total of 3600 (9 × 8 × 50) observations will be taken from 1 to 4 GHz for the computation purpose. Figure 9a represents the imaging setup with the head model and antenna element. The schematic profile and reflection coefficient of the antenna are also depicted with in Fig. 9b. The dimension is given in Table 3.

**Table 3:** Dimensions of the antenna prototype.

Parameter	Dimension (mm)	Parameter	Dimension (mm)	Parameter	Dimension (mm)
L	70	L8	4	f8	16
W	30	L9	3	m1	10
L1	3	f1	16	m2	1
L2	6	f2	20	m3	4.5
L3	17	f3	5	m4	2
L4	3.5	f4	4	m5	1.5
L5	3	f5	11	m6	0.5
L6	22.5	f6	6	A	10
L7	3	f7	4	B	10

The algorithm parameters will be working according to the key criteria for compatibility for the phantom imaging. First, the reflected signals, S(f, r_x_, φ) will be divided into two matrices including S_odd_ (f, r_x_, φ_odd_) and S_even_ (f, r_x_, φ_even_), where the values of φ_odd_ and φ_even_ are considered as φ_odd_ = 1,3,5,…Nφ − 1, and φ_even_ = 2,4,6,…Nφ, respectively. Therefore, S_odd_ and S_even_ are considered as initial and offset illumination. Next, the reflected signals will be converted into time-domain mode from the frequency domain mode using the Inverse Fourier Transform method that generates the Γ(t, rx, φ_odd_). Furthermore, the generated Γ(t, rx, φ_odd_) will be processed using the proposed IC-CF-DMAS method for reconstructing the images of imaging domain, where each point of these imaging domain are presented in Cartesian 3D coordinates from.

As the imaging domain is stationary, the position of the employed sensing antenna array needs to move from the reconstruction point. Then, the proper delay is generated by dividing the total distance with the background medium air and the dielectric constant is *ε*_*r*_.

1 $$\tau (i,r_{x} ,\varphi_{odd} ) = \frac{{\sqrt {\varepsilon_{r} } (p_{{C - Rx\varphi_{odd} }} (i,r_{x} ,l))}}{c}$$

here, *c* is the speed of light.

The delay is calculated from the anticipated smallest distance and the reflected signal from *C(i).* The delays are further added to the signals for delivering the proper delayed signal. After multiplying the paired delayed signal, they are summed for determining the scattering intensity at the allotted point in the region of interest, as shown in the following equation.

2 $$\Upsilon_{DMAS}^{{}} (i) = \int\limits_{ - \infty }^{\infty } {\sum\limits_{{\varphi_{odd} = 1}}^{{}} {\sum\limits_{rx = 1}^{{}} {\left[ {\Gamma (t - \frac{{\tau (i,r_{x} ,\varphi_{odd} )}}{\Delta t},r_{x} ,\varphi_{odd} )} \right]dt} } }$$

The CF-DMAS makes use of a weighted sum of the channels where the coherence factor (CF) will become modelled to incentive more coherent channels at every single stage in the imaging domain with more significant weights. The below equation computes it:

3 $$CF(i) = \frac{{\Upsilon_{DMAS} (i)}}{{\int\limits_{ - \infty }^{\infty } {\sum {_{{\varphi_{odd} }} \sum {_{rx} \Gamma \left( {t - \frac{{\tau (i,rx,\varphi_{odd} )}}{\Delta t},rx,\varphi_{odd} } \right)} } } dt}}$$

From then on, the scattering intensity map will be computed by the next formula:

4 $$\Upsilon_{CF - DMAS} (i) = CF(i).\Upsilon_{DMAS} (i)$$

The distance inverse weighting will be utilized for the reflection of the 3D Green function for electromagnetic waves.

5 $$\Upsilon^{^{\prime}} (i) = \int\limits_{C}^{{}} {\frac{{\Upsilon_{CF - DMAS}^{n - 1} (i)}}{{1 + p_{C - C} (i,j)}}} dj$$

After that, the modified delay will be estimated by the subsequent formula:

6 $$\tau^{^{\prime}} (i,rx,\varphi_{odd} ) = \tau (i,rx,\varphi_{odd} ) + \frac{{\Upsilon^{^{\prime}} (i)}}{c}$$

The Coherence Factor will be computed, and the scattering intensity map will be assessed as below:

7 $$\Upsilon_{CF - DMAS}^{n} (i) = CF(i).\Upsilon_{DMAS}^{n} (i)$$

In accordance with the modified delays, the scattering strength map will be reconstructed. Lastly, the closure requirements test for convergence. Equations (5)-(8) are assessed for n = 1 iteratively, 2…. 7.

8 $$E_{\Upsilon } = \sum {_{\forall i} } \left| {\Upsilon_{CF - DMAS}^{n} } \right. - \left. {\Upsilon_{CF - DMAS}^{n - 1} } \right|$$

**Figure 10:**
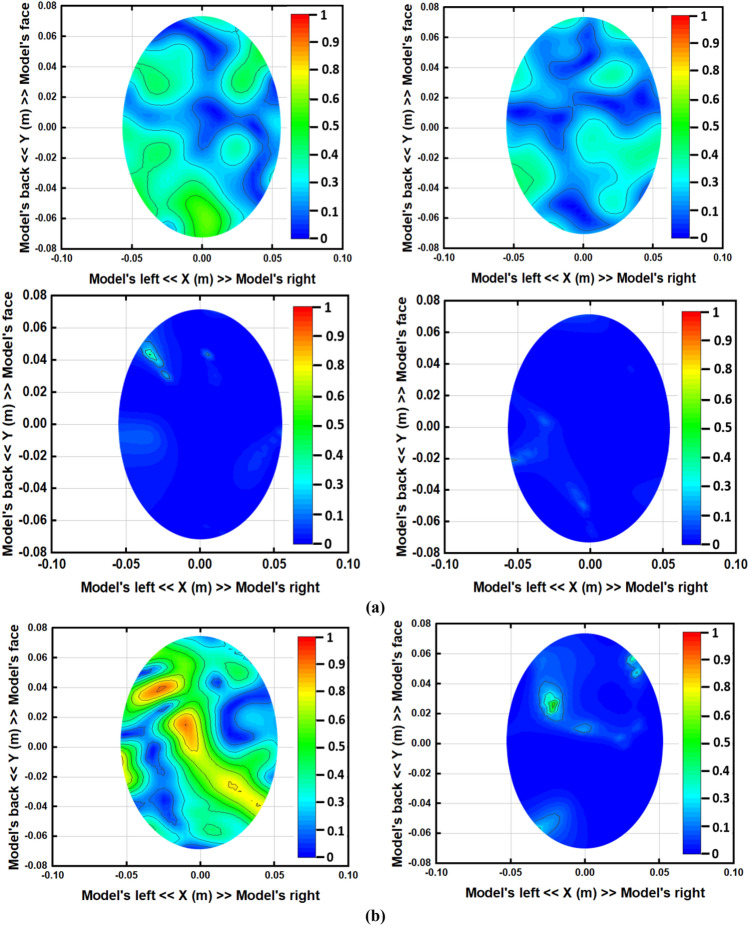

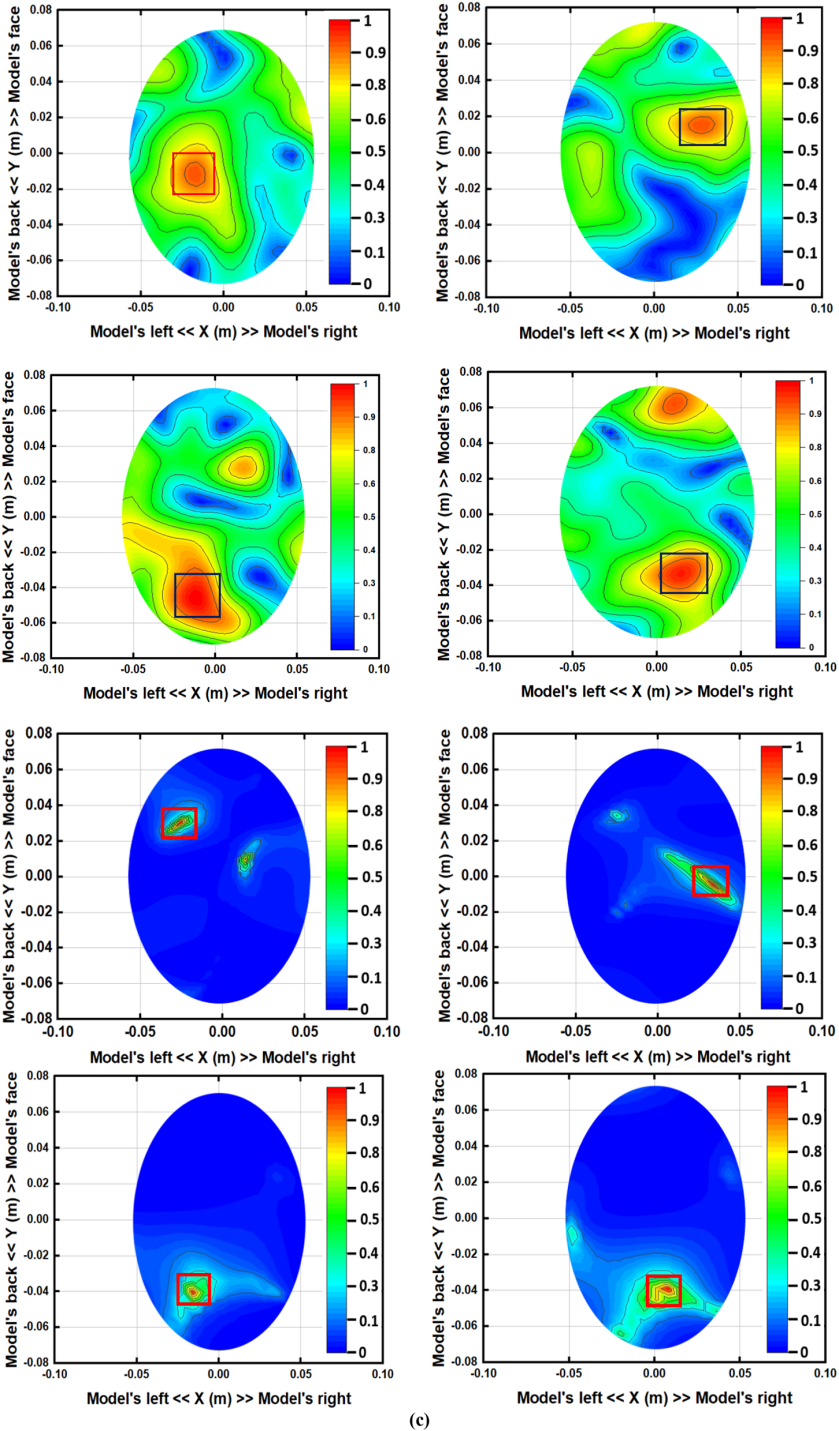
Reconstructed images using the antenna on different positions (with DMAS and IC-CF-DMAS) (**a**) blank data (**b**) human head model without stroke (**c**) human head model with stroke.

The iterative process will be prematurely terminated when Eϒ decreases to the preferred standard of precision as convergence has pretty much been realized. In this study, the iteration converged after seven trials and the error factor exponentially diminished with each iteration. This premature termination happens due to the limited execution time. Therefore, Eϒ < 10 − 5 is the convergence limit as per the analysis which will be applied to produce high quality and noiseless imaging data of an unhealthy phantom that will lead to efficient stroke detection performance. By differentiating the collected backscattered signals from the healthy and unhealthy tissue, the unhealthy tissue is identified as stroke, which can clearly be detected from this imaging analysis. Figure 10 represents the reconstructed images using the conventional DMAS and updated IC-CF-DMAS algorithm. Figure 10a illustrates the reconstructed blank images without the placement of the head model inside the system. The conventional DMAS and proposed IC-CF-DMAS have been utilized for the image reconstruction. It is noteworthy that the reconstructed images show very low noises within imaging domain. Figure 10b,c are the reconstructed images of the head model without and with stroke element. It is noticeable from the Fig. 10c that multiple highest contrast is formed within the imaging domain after applying the conventional DMAS algorithm that reflects the localization error. This occurs potentially due to the underestimation of the average dielectric constant of the imaging domain. Moreover, some “ghosting” or “halo effect” type distinct clutter is created due to the multiple reflections from the stroke object in conventional DMAS. This kind of clutter makes complications in detecting the actual stroke object. Furthermore, the error has been minimized by applying the proposed IC-CF-DMAS algorithm. The rectangular red mark presents the stroke detection and location. Four different stroke locations are used in the head model to examine and evaluate the antenna performance and reconstructed images. Significant noise reduction is noticed for the generated images. The IC-DMAS performs well by eliminating noise and ghosting and successfully detecting the stroke after removing localization errors.

## Stability in time

**Figure 11:**
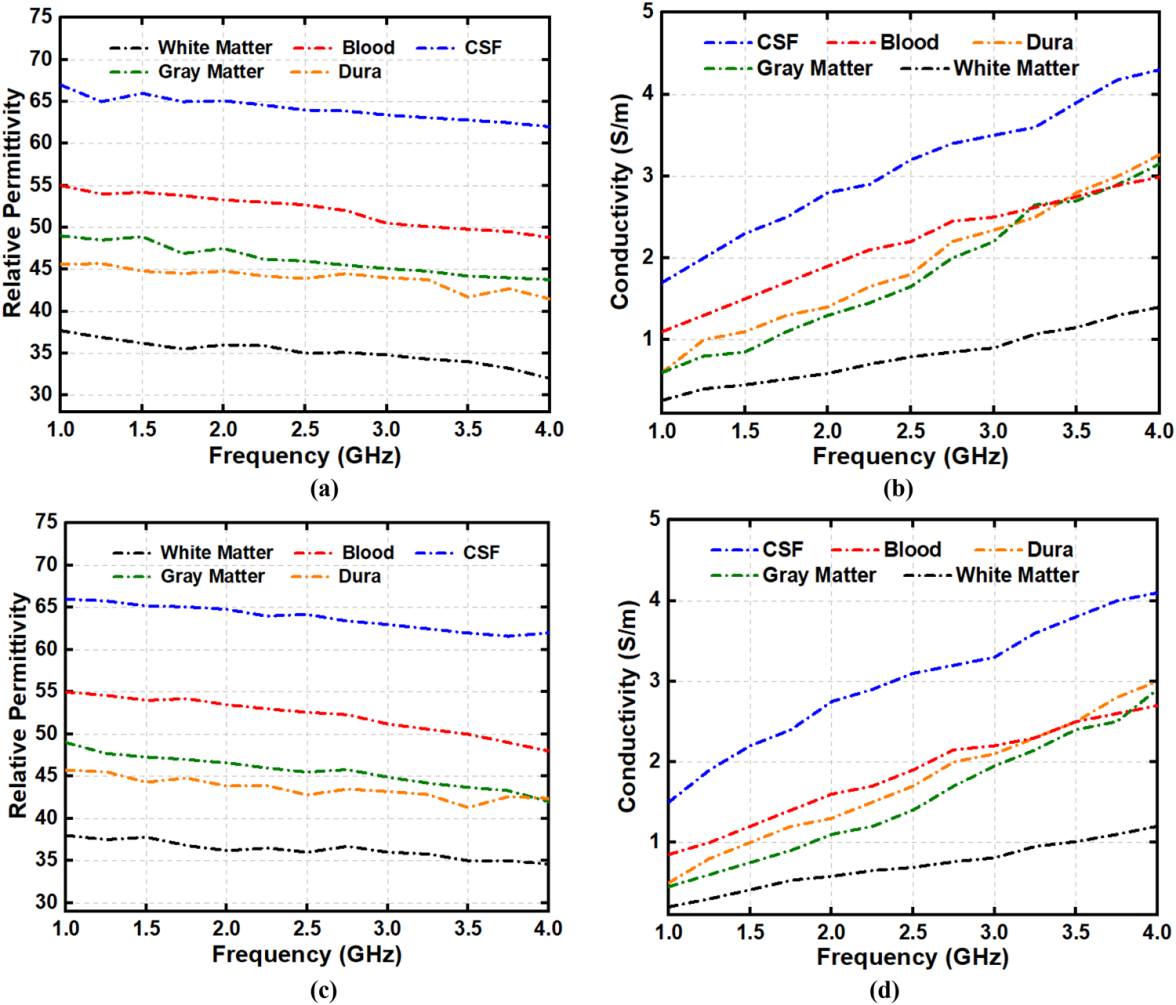
(**a**) Relative permittivity and (**b**) conductivity after 4 days; (**c**) relative permittivity and (**d**) conductivity after 7 days.

**Figure 12:**
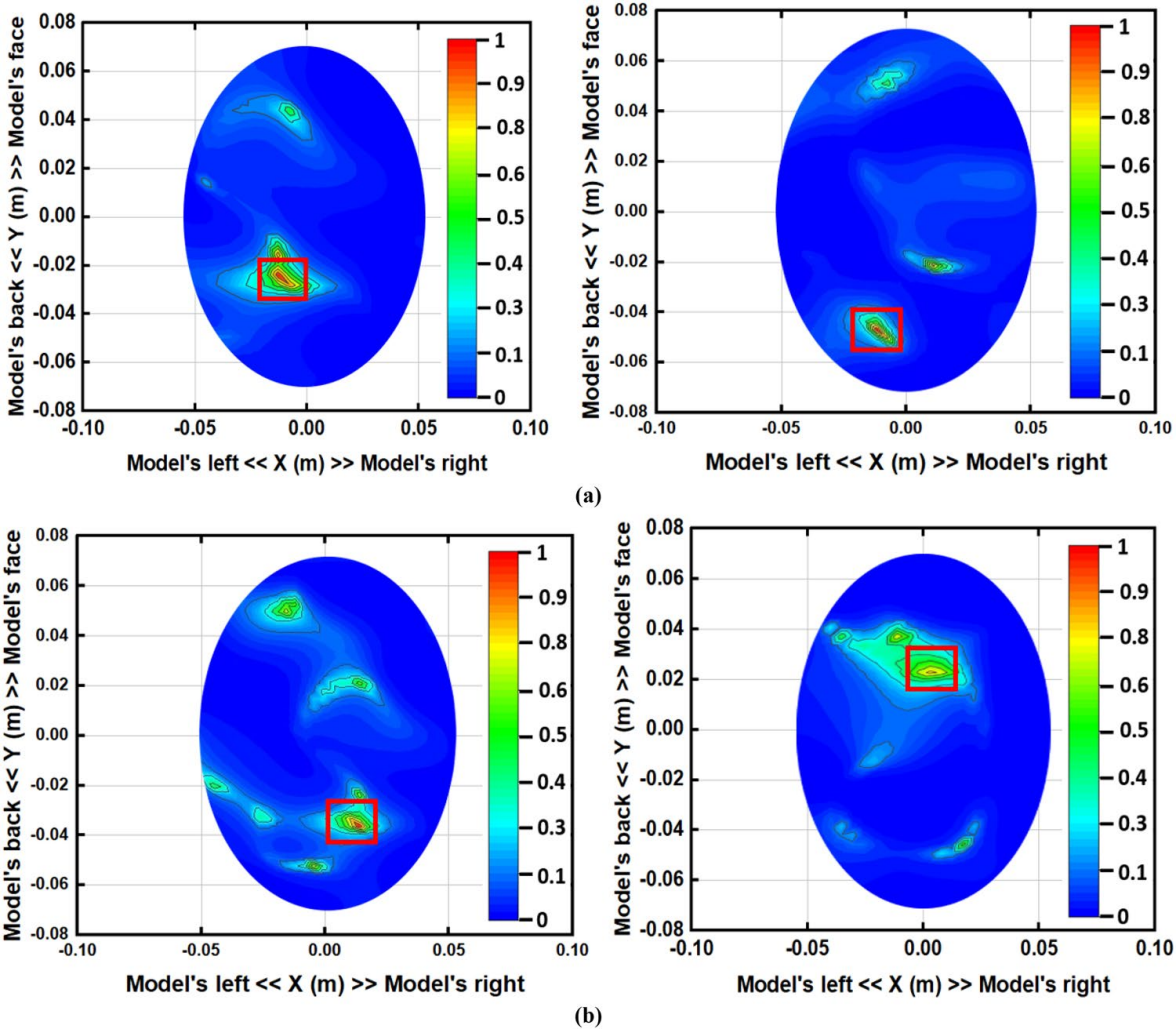
Reconstructed images of the human head model with stroke on different positions after (**a**) 4 days, and (**b**) 7 days.

It is important to evaluate the effectiveness of the fabricated tissue-mimicking head phantom as the brain’s tissue distribution is more complex towards the real imaging scenario. The fabricated phantom elements are kept for the second and third consecutive measurements after 4 days and 7 days within a week. The changes in the electrical properties are observed, and then the image reconstruction technique is performed to analyze the timely effects. The relative permittivity and conductivity of the fabricated phan-tom components are measured after 4 days, which is depicted in Fig. 11a,b. It is observing that there is a slight decrement occurred in electrical properties compared to the first day measurement. The decrement in measured electrical properties is also found after 7 days, which is presented in Fig. 11c,d. This happens due to the evaporation of water from the phantom components over time. As the phantom components need to be measured in a room temperature, it is expected to be evaporated over the time. Besides, preserving the phantom components below the freezing point can also make the water content evaporated. The better way to preserve the phantom components is to keep the phantom components in a less aired bag that reduces the evaporation process. Furthermore, the effectiveness of the phantom elements also depends on the image reconstruction results. The components are placed in the 3D skull after 4 days and 7 days to perform the image reconstruction process. The placement process is similar to Fig. 8. The reconstructed images after 4 days and 7 days are depicted in Fig. 12a,b. It is noticeable that the system can effectively identify the stroke from the phantom elements, although some noises are created due to the slight decrement of watery content from the phantom elements.

**Figure 13:**
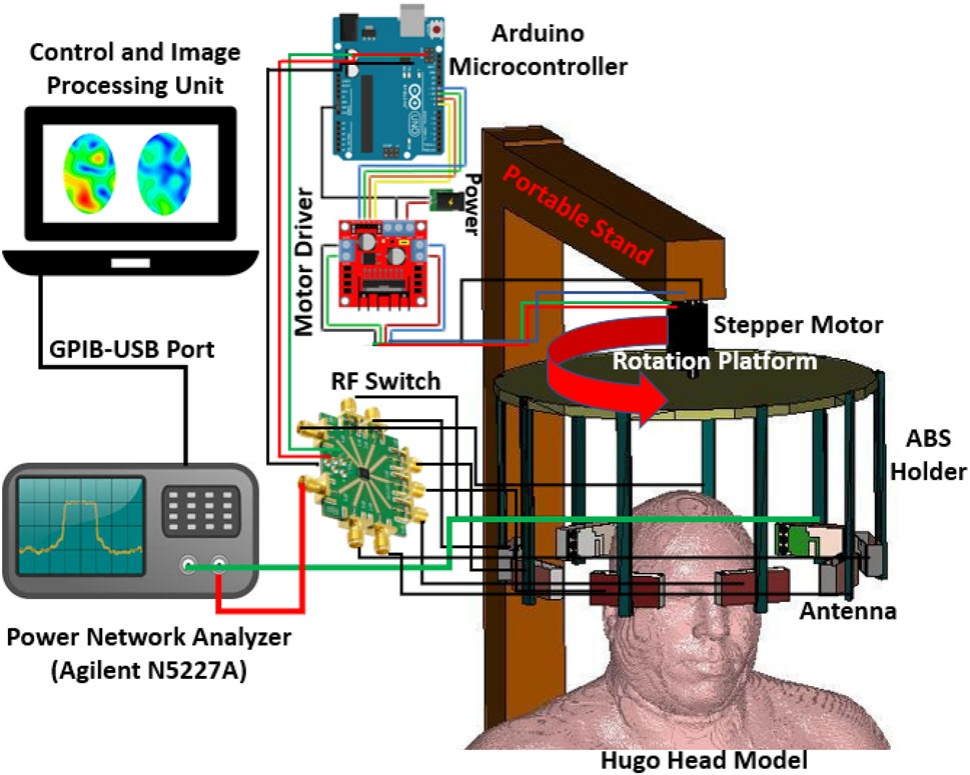
The schematic diagram of the portable EM head imaging system.

Table 4 represents the comparison of the developed tissue-mimicking head phantom and its detection capabilities with existing developed head phantoms. It is noteworthy from the analysis that the proposed tissue-mimicking head phantom has the maximum tissue elements with stability in time. Moreover, the proposed algorithm shows clear detection capabilities of the stroke with maximum number of tissue elements inside the head structure. Figure 13 represents the schematic diagram of the proposed portable EM head imaging system with its components for future real time installations. The height of the portable stand is 1.7 m, and the radius of the circular region is 180 cm. This proposed system will be suitable to be installed in the clinics and hospitals. The main components of the imaging system that need to be installed are power network analyzer, image processing unit and the portable imaging system.

**Table 4:** Comparison of developed tissue-mimicking head phantom with existing phantoms and detection capabilities. The bold in Table 4 is to highlight the properties of the proposed analysis compared to existing literature.

References	Frequency	Phantom type	Tissues	Structure	Stability in time	Imaging algorithm	Clear detection	Application
^26^	1.0 GHz	Liquid, homogeneous	Gray matter, white matter	Anthropomorphic	No	TSVD	Yes	Brain stroke imaging
^27^	1.1 GHz–1.6 GHz	Liquid, homogeneous	Scalp, skull, brain	Anthropomorphic	No	Not applied	Not applicable	Radiometric monitoring
^28^	2.4 GHz	Liquid, homogeneous	Human brain simulant	Anthropomorphic	No	Not applied	Not applicable	Hyperthermia
^29^	1.0 GHz-2.0 GHz	Semi-liquid, homogeneous	Gray matter, white matter, blood	Anthropomorphic	No	Huygens principle	Yes	Brain stroke imaging
^12^	1.0 GHz	Solid, multilayered	Scalp, CSF, brain	Layered	No	Not stated	Yes	Microwave tomography
^10^	1.0 GHz	Solid, multilayered	Scalp, CSF, brain, stroke	Layered	No	Not applied	Not applicable	Microwave stroke detection
^30^	2.0 GHz–5.0 GHz	Semi-solid, multilayered	Skin, bone, dura, gray matter, white matter	Stylized	No	Not applied	Not applicable	Implantable electronics
^31^	0.5 GHz–3.0 GHz	Semi-liquid, multilayered	CSF, brain, blood	ABS cavity	No	Not applied	Not applicable	Microwave imaging systems
^32^	1.0 GHz–6.0 GHz	Liquid mixtures, multilayered	Fat, brain, CSF	ABS cavity	No	Not applied	Not applicable	Microwave imaging systems
**Proposed**	**1.0 GHz–4.0 GHz**	**Semi-solid,** **heterogeneous**	**CSF, gray matter, white matter, dura, blood (Stroke)**	**Anthropomorphic (tissue-mimicking)**	**Yes**	**IC-CF-DMAS**	**Yes**	**EM head imaging system**

## Conclusion

A human head phantom preparation and fabrication based on the electrical properties within 1 to 4 GHz are presented in this paper. CSF, dura, white matter, gray matter, and blood/stroke are the five layers in head phantom that are prepared and fabricated as phantom elements. The measurement procedure is performed with the open-ended dielectric coaxial probe to evaluate the relative permittivity and conductivity. The IC-CF-DMAS algorithm is used to validate the head phantom and reconstruct the stroke images. The electrical properties and image reconstruction are performed again after 4 and 7 days to evaluate the effectiveness of the fabricated phantom elements. The system can effectively identify the stroke from the head phantom. Therefore, the head phantom is time-stable and suitable for testing the large data sets before transferring to the clinical trial.
